# PSYCHOLOGICAL AND HEALTH CONSEQUENCES OF HELPING OTHERS: INNOVATIVE METHODS TO UNDERSTAND STRAINS AND GAINS

**DOI:** 10.1093/geroni/igz038.872

**Published:** 2019-11-08

**Authors:** William E Haley, Karl Pillemer

**Affiliations:** 1 University of South Florida, Tampa, Florida, United States; 2 Cornell University, Ithaca, New York, United States

## Abstract

Older adults are often involved in prosocial behaviors including volunteering, informal assistance to family members, or extensive caregiving for family with chronic disease or disability. Many studies find that volunteering and providing informal support can enhance health and well-being, but family caregiving has generally been characterized as being highly stressful and harmful to health and well-being. Recent research has suggested that involvement in prosocial activities, including caregiving, can actually build resilience and buffer the impacts of stress, and that the commonalities across different types of prosocial behaviors in older adults deserve greater attention. This symposium brings together researchers who are using innovative methods to study prosocial behaviors, including measuring daily experiences and their linkages with affect, epidemiological methods, and use of health outcomes including serum biomarkers of inflammation and immunity, activity tracking, and mortality. Results across the presentations show that the effects of helping others can be considered as mixed blessings, with potentially harmful and helpful effects depending on contextual factors. Factors including a history of adverse childhood experiences, and dementia caregiving, can create particular challenges. The Discussant, Dr. Karl Pillemer, will discuss implications for future research on volunteering, informal assistance to family, and family caregiving. He will also address ways that gerontological researchers can present a more balanced public narrative about how stressful experiences such as caregiving can produce not only negative affect, but also potentially positive health benefits, resilience to stress, and personal growth.

